# Blocking meningeal lymphatic drainage aggravates Parkinson’s disease-like pathology in mice overexpressing mutated α-synuclein

**DOI:** 10.1186/s40035-019-0147-y

**Published:** 2019-03-01

**Authors:** Wenyan Zou, Tinglin Pu, Weixi Feng, Ming Lu, Ying Zheng, Renhong Du, Ming Xiao, Gang Hu

**Affiliations:** 1 Department of Pharmacology, School of Medicine and Life Sciences, Nanjing University of Chinese Medicine, 138 Xianlin Avenue, Nanjing, 210023 Jiangsu China; 2 Jiangsu Key Laboratory of Neurodegeneratiion, Department of Pharmacology, Nanjing Medical University, 101 Longmian Avenue, Nanjing, 211166 Jiangsu China

**Keywords:** A53T transgenic mice, α-Synuclein, Glymphatic clearance, Neurodegeneration, Parkinson’s disease

## Abstract

**Background:**

Abnormal aggregation of brain α-synuclein is a central step in the pathogenesis of Parkinson’s disease (PD), thus, it is reliable to promote the clearance of α-synuclein to prevent and treat PD. Recent studies have revealed an essential role of glymphatic system and meningeal lymphatic vessels in the clearance of brain macromolecules, however, their pathophysiological aspects remain elusive.

**Method:**

Meningeal lymphatic drainage of 18-week-old A53T mice was blocked via ligating the deep cervical lymph nodes. Six weeks later, glymphatic functions and PD-like phenotypes were systemically analyzed.

**Results:**

Glymphatic influx of cerebrospinal fluid tracer was reduced in A53T mice, accompanied with perivascular aggregation of α-synuclein and impaired polarization of aquaporin 4 expression in substantia nigra. Cervical lymphatic ligation aggravated glymphatic dysfunction of A53T mice, causing more severe accumulation of α-synuclein, glial activation, inflammation, dopaminergic neuronal loss and motor deficits.

**Conclusion:**

The results suggest that brain lymphatic clearance dysfunction may be an aggravating factor in PD pathology.

**Electronic supplementary material:**

The online version of this article (10.1186/s40035-019-0147-y) contains supplementary material, which is available to authorized users.

## Background

Parkinson’s disease (PD) is an age-dependent neurodegenerative disease, characterized by progressive loss of midbrain dopaminergic neurons and formation of Lewy bodies [1, 2]. Although the pathogenesis of PD has not been completely elucidated, excessive accumulation of toxic forms of α-synuclein (α-syn) with a series of secondary pathological cascades plays crucial roles in the onset of PD. This is mainly due to an imbalance between production and clearance of α-syn in the brain [3, 4]. A small part of patients with early-onset PD show increased α-syn production because of mutations in the α-syn gene, while the vast majority of PD patients suffer from decreased removal of α-syn from the brain [5, 6]. Previous studies have reported that α-syn is degraded through various intracellular clearance mechanisms including autophagy lysosomal pathways [7–9]. However, it is unclear whether there is an extracellular route to remove soluble α-syn directly from the brain. Exploring this issue will help to discover potentially new strategies for the prevention and treatment of PD.

Recent findings have suggested that brain lymphatic drainage system including a brain-wide network of paravascular channels, termed the ‘glymphatic system’ and meningeal lymphatic vessels drain macromolecules from the brain parenchyma to the deep cervical lymph nodes (Dclns) [10, 11]. Further evidence suggests that glymphatic function depends on astroglial aquaporin-4 (AQP4), contributing to a major portion of brain soluble amyloid-β (Aβ) clearance [12]. Impaired glymphatic clearance exacerbates Aβ deposition in the brain of a mouse model of Alzheimer’s disease (AD) [13]. In addition, the glymphatic system facilitates convective exchange of various interstitial solutes including glucose, lipids, amino acids, lactic acid and neurotransmitters between cerebrospinal fluid (CSF) and interstitial fluid (ISF) [14, 15]. Furthermore, disruption of meningeal lymphatic drainage aggravates parenchymal Aβ accumulation and cognitive decline of AD transgenic mice [16, 17]. However, it remains unclear whether glymphatic clearance dysfunction is involved in brain α-syn aggregation and related pathology.

To address this question, we investigated glymphatic clearance function of A53T mice overexpressing mutated human α-syn. The consequence of blocking meningeal lymphatic drainage on α-syn-related pathophysiology was also observed in the PD animal model. The finding suggests that impaired brain lymphatic clearance is involved in PD pathology.

## Materials and methods

### Animals

A53T mice were constructed by Nanjing University [18]. Age-matched wild-type (WT) mice were used as controls. The α-syn cDNA content of homozygous A53T mice was more than 2-fold higher than that in the controls as determined by quantitative PCR. Genotyped male mice were housed under standard conditions (room temperature 18 ~ 22 °C, humidity 30 ~ 50%, well-ventilated, a 12-h light-dark cycle). In addition, three-month old male AQP4 knockout (AQP4^−/−^) mice in a CD1 genetic background [19] were used to evaluate the effect of Aqp4 gene deletion on removal of injected exogenous α-syn clearance from the brain parenchyma. All efforts were made to minimize and reduce number of animals used. The animal experiments were approved by the Institutional Animal Care and Use Committee of Nanjing Medical University.

### Ligation of the deep cervical lymph nodes (LDclns)

The procedure of LDclns was performed according to published literature [11]. Briefly, after anesthetized with 4% chloral hydrate, 18-week-old A53T mice and WT mice were fixed on a stereotaxic apparatus in a supine position, and longitudinally incised along the midline of the neck (approximately 1 cm). Under a microscope, fat and soft tissue were separated with a blunt forceps. Then, the sternocleidomastoid muscles were retracted and both sides of the dCLNs were exposed. Their afferent vessels were carefully ligated using 8–0 nylon suture. The mouse skin was sutured and disinfected with iodophor. Sham-operated mice were only surgically exposed the dcLNs without ligation. The animals were returned to their cages after recovery from anesthesia and continued to be fed for 6 weeks, followed by behavioral testing or CSF tracer experiments.

### Open-field test

The experiment was used to assess autonomic motor ability in mice [20]. Mice were placed in an open box (length: 60 cm, width: 60 cm; height: 25 cm) and allowed to move freely for 5 min. The total distance traveled was recorded by an open field software (Clever Sys Inc., VA, USA). After each test, the box was cleaned for avoiding the odors that might affect testing results of the next animal.

### Rotarod test

Rotarod test was used to detect the ability of motor coordination on a rotating rod (diameter 3.2 cm) [21]. Mice were trained for 5 min, 3 times a day for 3 days. The speed started at 4 rpm and accelerated at 0.1 rpm/s. The test was performed three times per mouse, and the time of mouse falling from the rotarod was recorded. The mean time for 3 independent experiments was then calculated.

### Pole test

Pole test was used to evaluate autonomic ability of mice [22]. A straight wooden bar of 1 cm in diameter and 50 cm in length was made. The top of the stick was fixed with a wooden ball (about 1.2 cm in diameter). The ball was wrapped around the gauze to prevent the animal from slipping off. The mouse was placed on the top of the rod, the time for completing turn of the head (T-turn) and the total time from the top to the bottom of the rod (T-total) were recorded. If the mouse failed to flip or slip completely, the time was recorded as 120 s. All mice were trained three times per day for three days prior to the formal testing.

### Intracisternal infusion of fluorescent tracer

Intracisternal infusion of fluorescent tracer was performed as previously described [13]. Anesthetized mouse was fixed in a stereotaxic instrument, and the posterior atlanto-occipital membrane was exposed. Five microliter of Texas Red-dextran-3 (TR-d3, MW 3 kD; Invitrogen; Cat # D3328) at 0.5 mg ml^− 1^ in artificial CSF was infused into the cisterna magna through a 50 μl syringe mounted with a 27-gauge needle, connected to a constant current syringe pump. The intracisternal infusion was carried out at a rate of 2 μl/min. Thirty min after the start of infusion, the mouse was anesthetized again and perfused trans-cardially with 4% paraformaldehyde (PFA). The brains and dcLNs were removed and post-fixed in the same fixative for 24 h.

### Stereotaxic injection of α-syn

AQP4^−/−^ mice and WT mice were given stereotaxic injection of soluble recombinant human α-syn. An injection cannula (27 gauge) was inserted stereotaxically into substantia nigra (SN) (anteroposterior: − 3.0 mm; mediolateral: ±1.3 mm; dorsoventral: − 4.2 mm) [23]. The infusion was performed at a rate of 0.25 μl/min, and 2.5 μl of recombinant human α-syn Ala30-Pro (A30P) or α-syn A53T (Sigma-Aldrich; Cat # S1196; Cat # S1071; 3 μg/ml in phosphate buffer solution (PBS)), or the same volume of PBS were injected into the mouse. At 30, 60 or 120 min after injection, mice were sacrificed for enzyme-linked immunosorbent assay (ELISA) to detect injected α-syn residue in the brain. Western blotting and immunofluorescence were also performed on ventral midbrain samples at the time point of 120 min.

### Brain water content measurement

Mice were anesthetized and brains were removed, weighed, heated in an oven at 70 °C for 72 h and reweighed. Percentage water content was calculated as [(wet weight–dry weight)/wet weight × 100%] [24].

### Section preparation

For section preparation, all mice were anesthetized with 4% chloral hydrate, and perfused with saline for 3 min followed by 4% PFA for 5 min. Brain tissues and Dclns were fixed in 4% PFA overnight after perfusion and dehydrated with 20% sucrose solution dissolved in 0.01 M PBS for 3 days. 30 μm slices were cut coronally throughout the midbrain on a cryostat (Leica, Wetzlar, Germany) and mounted onto gelatin-coated slides. The lymph nodes were also cryostat sliced at 30 μm. For CSF tracer experiments, PFA post-fixed midbrain tissues were sliced on a vibratome (Leica) at 100 μm and mounted onto gelatin-coated slides in sequence.

### Cerebral dura mater stripping

The skull was separated into the upper half and lower half utilizing microsurgical scissors. A complete skull obtained after brain removed was placed in 20% sucrose for 3 days and then transferred into sterilized water for 2 days. Under a stereomicroscope, the dura mater was carefully dissected from the skull using a pair of ophthalmic forceps, then unfolded on glass slide covered with poly-l-lysine staining.

### Hematoxylin-eosin (H&E) staining

Lymph nodes were stained with hematoxylin solution for 5 min and washed with distilled water for 1 h, then placed in 70 and 90% alcohol for 10 min, respectively. The sections were stained with Eosin solution for 2–3 min, dehydrated with pure alcohol, transparented with xylene, and sealed by neutral gum.

### Immunohistochemistry

Immunohistochemical staining was performed as previously described [25]. The sections were incubated overnight at 4 °C with the following primary antibodies: mouse anti-tyrosine hydroxylase (TH, 1:4000, Sigma-Aldrich, Cat # T1299), mouse anti-glial fibrillary acid protein (GFAP, 1:1000, Millipore, Cat # MAB360) and rabbit anti-ionized calcium binding adapter molecule 1 (Iba-1, 1:1000, Wako, Cat # 019–19,741). After rinsing with PBS, the sections were incubated with biotinylated goat anti-mouse or rabbit IgG (11000) for 1 h at room temperature and thereafter incubated with streptavidin-biotin-peroxidase complex (Elite ABC Kit, Vector Laboratories, Cat # PK-7200) and visualized with 2,2′-diaminodiphenyl.

### Immunofluorescence

Brain slices were blocked for 1 h at room temperature with 5% bovine serum albumin, incubated with a mixture of mouse anti-α-syn (1:1000, BD Transduction Laboratories, Cat # 610787) and rabbit anti-AQP4 (1:300, Millipore, Cat # AB3594), rabbit anti-laminin (1:300, Sigma-Aldrich, Cat # L9393) or rabbit anti-TH (1:500; Sigma-Aldrich, Cat # SAB4502964), or mouse anti-CD31 (1:500, Sigma-Aldrich, Cat # P8590) and rabbit anti-AQP4. Alexa Fluor 488 and Alexa Fluor 594 secondary antibodies (Thermo Fisher Scientific, 1:1000, Cat # A-11034; Cat # A-11032) were incubated for 1 h at room temperature. Meninges were incubated with a monoclonal mouse anti-lymphatic vessel endothelial hyaluronan receptor 1 (lyve-1) antibody (1:1000, Abcam, Cat # ab33682) and thereafter incubated with Alexa Fluor 488 secondary antibody.

### Cell counting and image analysis

Immunohistochemically stained sections were observed by an Olympus BX52 microscope (Olympus America Inc., Melville, NY, USA) with an optical fractionator (Stereo Investigator 7, MBF bioscience, Williston, VT, USA). The numbers of TH-, GFAP- and Iba-1-positive cells in SN were assessed as described previously [26]. Briefly, regions of each side of SN were outlined at low magnification (40×). The counting frame size was 50 μm × 50 μm and the sampling grid size was 100 μm × 100 μm. The sampling scheme was designed to have coefficient of error (CE) less than 10% in order to get reliable results. The number of immunoreactive cells mentioned above was counted on 13 serial sections per mouse respectively. The data were presented as the mean of 4 mice in each group. Percentage of GFAP or Iba-1P positive area was also counted by NIH Image J.

The midbrain sections with immunofluorescence and CSF fluorescence tracer were photographed by a digital microscope (Leica Microsystems, Wetzlar, Germany). The image was analyzed using Image J and fluorescence signal of AQP4, α-syn and TR-d3 was measured respectively, by using the interest grayscale threshold analysis [27]. Percentage of fluorescence signal positive area in midbrain was calculated. The AQP4 polarization in SN was obtained by the ratio between the AQP4 fluorescence intensity of the perivascular domains and the adjacent neutrophil domains [27, 28]. Each index was averaged from 6 to 8 midbrain sections per mouse in each group of 4 mice. The images were acquired and analyzed by a double-blind investigator.

### Brain homogenate preparation

Mice were anesthetized, and ventral midbrain was quickly dissected out, isolated, weighed and crushed. Radio immunoprecipitation assay (RIPA) protein lysate was then added at a mass/volume ratio of 1:10 (10 μl/1 mg) and lysed on ice for 30 min at 4 °C, and centrifuged 15 min at 16000 rpm [24]. To analysis insoluble α-syn oligomeric protein, 5 volumes of Triton X-100 insoluble lysate were added to the precipitated protein obtained from centrifugation, sonication, boiling at 95 °C for 20 min, and centrifuged again for additional 10 min at room temperature [29]. Protein concentration of the samples was determined by a bicinchoninic acid protein assay kit (Kevgen Biotech, Cat # ab6672). Bovine). Bovine serum albumin (1 mg/ml) was used as standard protein.

### Western blotting

The brain samples were transferred onto polyvinylidene fluoride membranes using a Bio-Rad miniprotein-III wet transfer unit, followed by blocking with 5% nonfat milk dissolved in Tris-buffered saline with 0.1% Tween 20 (TBST) at room temperature for 1 h [24]. Membranes were then probed with the following primary antibodies: mouse anti-β-actin (1:1000, Sigma-Aldrich, Cat # A2228), mouse anti-TH (1:4000, Sigma-Aldrich Cat # T1299), mouse anti-α-syn (1:1000, BD Transduction Laboratories, Cat # 610787), goat anti-interleukin 1β (IL-1β; 1:1000, Sigma-Aldrich; Cat # I3767), rabbit anti-IL-6 (1:1000, Abcam, Cat # ab6672), rabbit anti-tumor necrosis factor-α (TNF-α; 1:800, Abcam, Cat # ab9739), rabbit anti-light chain 3B (LC3B, 1:1000, CST, Cat # 2775), rabbit anti-p62 (1:500, Abcam, Cat # ab56416), rabbit anti-tau (1:1000, Abcam, Cat # ab32057) or rabbit anti-phosphorylated Tau Ser396/Ser404 (PHF-1, 1:1000, Abcam, ab184951) then followed by overnight incubation at 4 °C. The blots were incubated with horseradish peroxidase (HRP)-conjugated secondary antibodies at room temperature for 1 h, and signals were detected by chemiluminescence (ECL, Pierce, USA). Images were acquired with Image Quant LAS 4000 mini (GE Healthcare, USA) and analyzed using Image J. Analyses were completed in duplicate and the mean value was calculated.

#### Elisa

Soluble and insoluble α-syn from ventral midbrain samples were quantified using a commercial ELISA kit (Abcam, Cat # ab210973). The assay procedure was carried out according to the manufacturer’s instructions. Briefly, samples or standards were added to the 96-well plate and mixed with the antibody. After incubation, the wells were washed to remove unbound material. 3,3′5,5′-tetramethylbenzidine (TMB) substrate was added and incubation was catalyzed by HRP to generate blue colorimetric reaction. The reaction was stopped with 100 μl 1 M HCl and absorbance was read at 450 nm using a 96-well microplate reader (GE Healthcare, USA). All measurements were performed in duplicate and the mean value was calculated.

### Flow cytometry

Mice were perfused from the left cardiac ventricle with cold PBS for 10 min. Meninges were dissected with fine forceps and digested in RPMI-1640 medium (Sigma-Aldrich, Cat # R0883) + 1.4 U/ml Collagenase VIII (Sigma-Aldrich, Cat # C2139) + 1 mg/ml DNase1 (Sigma-Aldrich, Cat # 11119915001) for 15 min at 37 °C [11]. Dclns were removed and smashed in dulbecco’s modified eagle’s medium (DMEM) containing 10% fetal calf serum. Digested meninges and smashed Dclns were dissociated using pipetting, and were passed through 70-μm filters. Cell suspension was incubated with antibody dye CD3 (1:400, eBioscience, Cat # 145-2C11), CD4 (1:400, eBioscience Cat # 17–0041-83) or CD8 (1:400, BD Bioscience Cat # 335787) for 30 min at 4 °C. Data were acquired on an LSRII cytometer (BD Biosciences, Franklin Lakes, NJ, USA) and analyzed using the Flowjo Pro software (FlowJo, LLC, Ashland, OR, USA). The number of cells used for each analysis was 10,000 for the meningeal samples and 50,000 for the Dclns. Mean value of each sample was obtained from three independent experiments.

### Statistical analysis

All data were expressed as means ± SEM. Statistical analyses were performed with Prism 6 (Graph-Pad, La Jolla, CA, USA). Data were analyzed by two-way analysis of variance (ANOVA) followed by Tukey’s post-hoc multiple comparison test or Student-t test as indicated in the figure legends. *p* < 0.05 was considered to have statistical significance.

## Results

### LDclns blocks meningeal lymphatic drainage

**Fig. 1:**
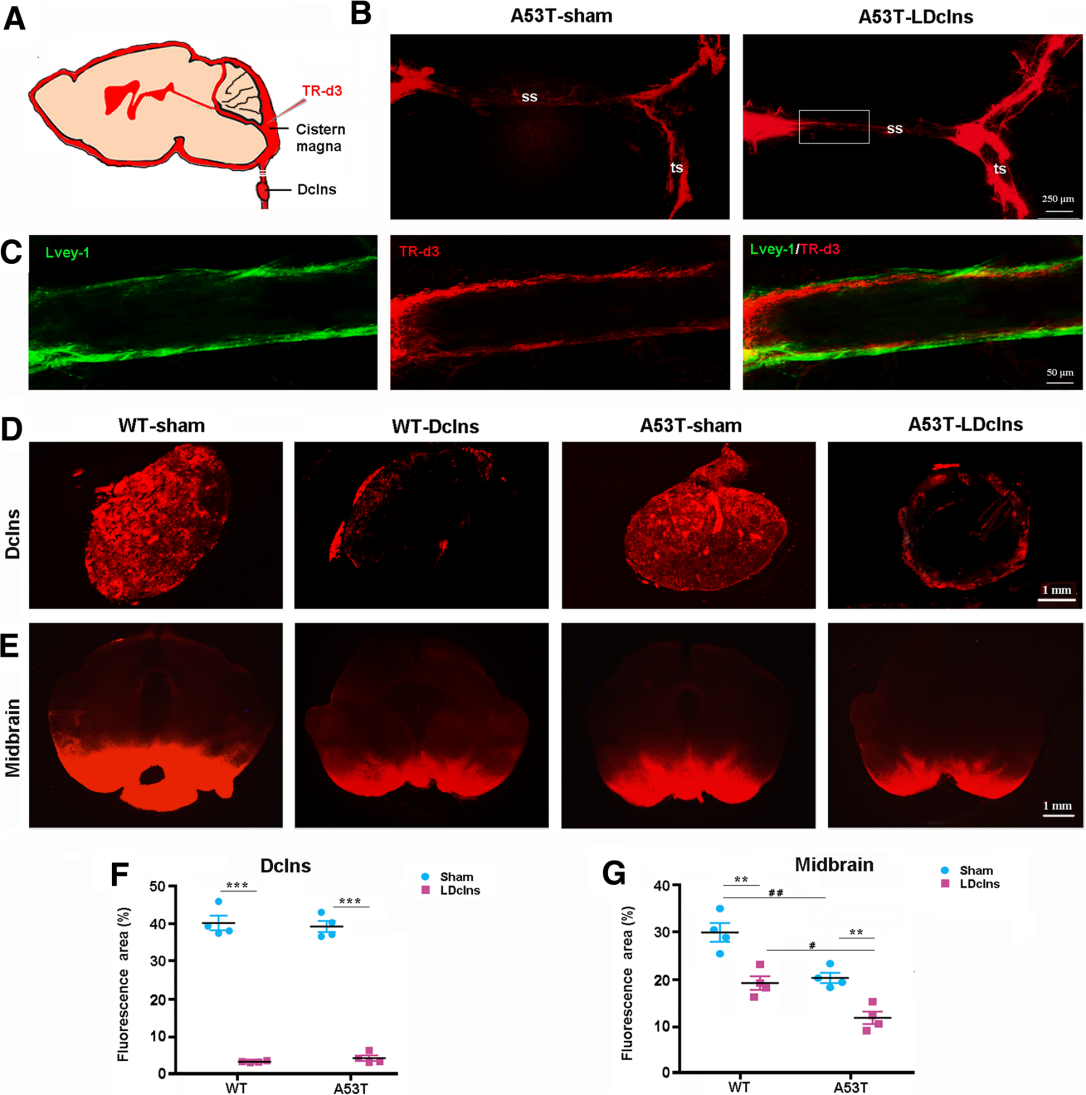
LDclns blocked peripheral drainage of intracisternal TR-d3 and further reduced TR-d3 influx into brain parenchyma of A53T mice. **a** Schematic diagram showing injection of TR-d3 into the cisterna magna. **b** Representative images of TR-d3 fluorescence within superior sagittal (ss) and transverse (ts) meningeal lymphatic vessels of A53T-sham mice and A53T-LDclns mice. LDclns enhanced filling of dural lymphatic vessels. **c** High magnification micrographs showing that the fluorescent dye was restrictively within Lvey-1 positive lymphatic vessels. **d**-**e** Representative images showing TR-d3 fluorescence within the Dclns (**d**) and midbrain (**e**) at 30 min after injection into the cisterna magna. **f** Percentage of TR-d3 fluorescent area in the Dclns (genotype, F_(1,12)_ = 1.739, *p* = 0.2119; ligament, F_(1,12)_ = 75.13, *p* < 0.0001; interaction, F_(1,12)_ = 0.7534, *p* = 0.4024). **g** Percentage of TR-d3 fluorescent area in the midbrain (genotype, F_(1,12)_ = 32.28, *p* = 0.0001; ligament, F_(1,12)_ = 41.13, *p* < 0.0001; interaction, F_(1,12)_ = 0.5650, *p* = 0.4668). Statistical analysis was performed by Two-way ANOVA followed by Tukey’s post-hoc test. * *p* < 0.01, ** *p* < 0.01, *** *p* < 0.001, Ldclns vs Sham; #*p* < 0.05, ## *p* < 0.01, ### *p* < 0.001, A53T vs WT. Unless otherwise noted, the statistical method is also analyzed the data in Figs. 2-8

We first verified whether LDclns would block meningeal lymphatic drainage by injection of fluorescent tracer TR-d3 into the cisterna magna of mice (Fig. 1a). TR-d3 was observed in Lvey-1 positive dural lymphatic vessels at 30 min after injection, and was almost fill up the transverse dural lymphatic vessels after LDclns (Fig. 1b, c). TR-d3 fluorescent signal was distributed throughout Dclns in sham-operated mice, while only a small amount of fluorescent dye penetrated into the marginal portion of ligated Dclns (Fig. 1d). Quantitative analysis showed that percentage of TR-d3 positive area in Dclns of ligated mice was only ~ 5% of the sham controls (Fig. 1f). In addition, H&E staining demonstrated that ligated Dclns underwent atrophy and a considerable number of lymphatic cells were lost and replaced by fibroblast-like cells (Additional file 1: Figure S1). We also determined whether LDclns disrupted meningeal lymphatic drainage of immune cells into the peripheral lymph system by flow cytometry analysis of T cell subsets. The number of CD3^+^, CD4^+^ and CD8^+^ T cells significantly increased in the meninges, and decreased in the Dclns in both WT and A53T ligated mice (Additional file 2: Figure S2a-d). Together, these results revealed that meningeal lymphatic drainage was chronically disrupted after LDclns.

### LDclns decreases glymphatic influx of CSF tracer

We then observed interactive effects of LDclns and α-syn overexpression on glymphatic transport function of mice. After injecting TR-d3 into the cisterna magna for 30 min, fluorescent signal of TR-d3 was clearly observed in the whole SN region of WT-sham mice, but the fluorescence intensity was reduced in the other three groups, especially, in A53T-LDclns mice (Fig. 1e). The percentage of TR-d3 diffusion area into the midbrain of WT-LDclns mice, A53T-sham mice and A53T-LDclns mice was 65, 67 and 42% of WT-sham mice respectively (Fig. 1g).

### LDclns exacerbates α-syn aggregation in A53T mouse brain

**Fig. 2:**
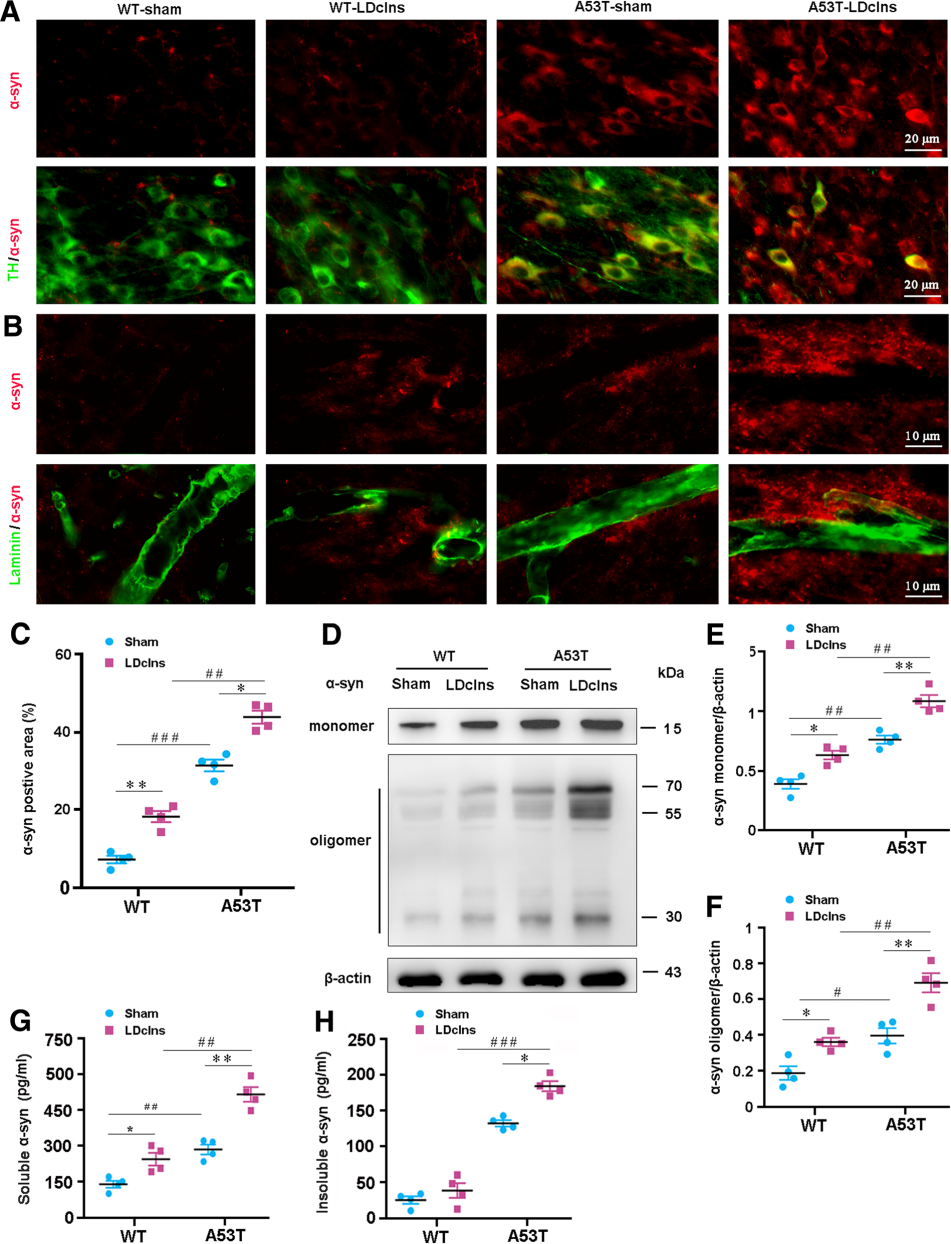
LDclns increased intracellular and extracellular aggregation of α-syn in SN of A53T mice. **a** Representative images showed that α-syn positive intercellular inclusion was increased in TH positive neurons of Ldclns-A53T mice. **b** Extracellular aggregation of α-syn in adjacent region of laminin-positive microvessels in both WT-LDclns mice and A53T mice, and further increased in A53T-Ldclns mice. **c** Percentage of α-syn positive area in SN was higher in A53T-LDclns mice than A53T-sham controls (genotype, F_(1,12)_ = 67.8, *p* < 0.0001; ligament, F_(1,12)_ = 50.39, *p* < 0.0001; interaction, F_(1,12)_ = 2.684, *p* = 0.1273). **d**-**f** Representative blotting bands and densitometry analysis of α-syn monomer and oligomers (monomer: genotype, F_(1,12)_ = 49.07, *p* < 0.0001; ligament, F_(1,12)_ = 25.97, *p* = 0.0003; interaction, F_(1,12)_ = 0.1873, *p* = 0.6729. oligomers: genotype, F_(1,12)_ = 43.24, *p* < 0.0001; ligament, F_(1,12)_ = 32.94, *p* < 0.0001; interaction, F_(1,12)_ = 2.234, *p* = 0.1608). **g** ELISA analysis of soluble α-syn from ventral midbrain samples (genotype, F_(1,12)_ = 47.01, *p* < 0.0001; ligament, F_(1,12)_ = 40.22, *p* < 0.0001; interaction, F_(1,12)_ = 1.781, *p* = 0.2068). **h** ELISA analysis of insoluble α-syn from ventral midbrain samples (genotype, F_(1,12)_ = 76.2, *p* < 0.0001; ligament, F_(1,12)_ = 28.07, *p* = 0.0002; interaction, F_(1,12)_ = 4.115, *p* = 0.0653). Data represent mean ± SEM from 4 mice per group; data in **f** is from two independent experiments and **g**-**h** from three independent experiments

Based on impaired glymphatic solute transport, we investigated whether LDclns improved aggregation of endogenous α-syn in the brain interstitium of WT and A53T mice. There was intracellular and extracellular aggregation of α-syn in SN of A53T mice, which was exacerbated following LDclns, as revealed by double immunofluorescence for α-syn and TH (Fig. 2a) or laminin (Fig. 2b). Mild accumulation of brain α-syn was also observed in WT-LDclns. Quantitative data revealed that α-syn immunofluorescent area of A53T-LDclns mice was larger than that of A53T-sham mice, indicating more worsened glymphatic clearance dysfunction (*p < 0.05;* Fig. 2c). Consistently, Western blotting showed that under the basal conditions, the levels of soluble α-syn monomeric protein and insoluble α-syn oligomeric protein in ventral midbrain of A53T mice were higher than those in WT control mice (*p* < 0.05; *p* < 0.001, respectively), and these differences were further increased after LDclns (both *p* < 0.05; Fig. 2d-f). We also examined the effect of LDclns on ISF levels of α-syn using ELISA. Results showed that the level of soluble α-syn in ventral midbrain of both genotype mice was markedly increased via blocking meningeal lymphatics (*p* < 0.05, WT-LDclns vs WT-sham; *p* < 0.01, A53T-LDclns vs A53T-sham; Fig. 2g), and the level of insoluble α-syn was only increased in A53T mice (*p* < 0.05, A53T-sham vs A53T-LDclns; Fig. 2h). Potentially, this was mainly due to an extremely small amount of insoluble forms of α-syn in WT mouse brain (*p* < 0.001, A53T-sham vs WT-sham; *p* < 0.001, A53T-LDclns vs WT-LDclns).

**Fig. 3:**
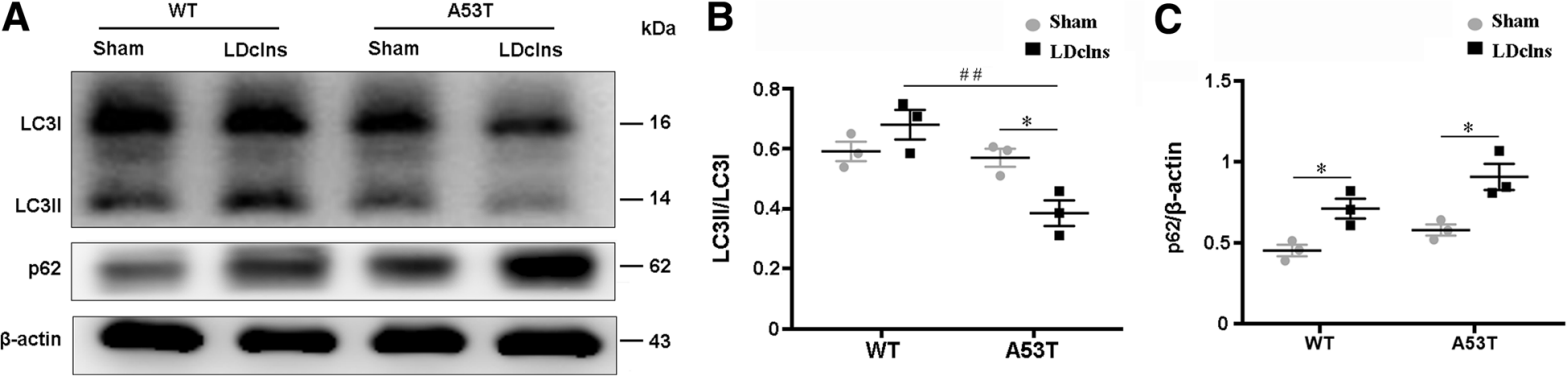
LDclns inhibited autophagy in ventral midbrain of A53T mice. **a**-**c** Representative immunoblotting bands and densitometry analysis of LC3 and p62. The ratio of LC3II/LC3I was significantly decreased in A53T-LDclns mice (genotype, F_(1,8)_ = 16.10, *p* = 0.0039; ligament, F_(1,8)_ = 1.481, *p* = 0.2583; interaction, F_(1,8)_ = 12.09, *p* = 0.0084). LDclns increased levels of p62 in both WT and A53T mice (genotype, F_(1,8)_ = 8.138, *p* = 0.0214; ligament, F_(1,8)_ = 26.94, *p* = 0.0008; interaction, F_(1,8)_ = 0.3773, *p* = 0.5561). Data represent mean ± SEM from 3 mice per group from two independent experiments

Previous literature reported that autophagy participates in intracellular degradation of α-syn and is impaired in A53T mice [30, 31]. We determined whether LDclns aggravated autophagy dysfunction in A53T mice. Western blotting revealed a down-regulation of autophagy improving marker LC3II/LC3I and an up-regulation of autophagy inhibitory marker p62 in ventral midbrain of A53T-LDclns mice (Fig. 3a-c). This suggests that impaired glymphatic clearance pathway and inhibited autophagy together contribute to excessive aggregation of α-syn within A53T mice, and worsen after LDclns.

We also analyzed whether LDclns would affect clearance of other macromolecules from the brain. Results showed that protein levels of total Tau and PHF-1, one type of phosphorylated Tau, were higher in A53T-LDclns mice than those in WT-LDclns mice and A53T mice (all *p* < 0.05; Additional file 3: Figure S3a-c). This result is consistent with the view that there is a synergistic interaction between α-syn and tau in mediating neurodegeneration in PD, as α-syn may increase tau aggregation [32–34] and treating A53T mice with tau oligomer specific monoclonal antibody can alleviate PD-like pathophysiological phenotypes [35].

In addition, we examined whether blocking meningeal lymphatics affects clearance of excessive ISF from the brain. Brain water content was not different between WT mice and A53T mice with or without LDclns (Additional file 4: Figure S4). These data are consistent with a pervious study showed that lack of dural lymphatic vessels compromises CNS macromolecule clearance, but does not affect CNS water homeostasis [11]. This might be due to a compensatory role of other water transport mechanisms including transport across the blood-brain-barrier and absorption at the arachnoid granulations [11, 15].

### LDclns exacerbates reactive gliosis and inflammatory cytokine production in SN of A53T mice

**Fig. 4:**
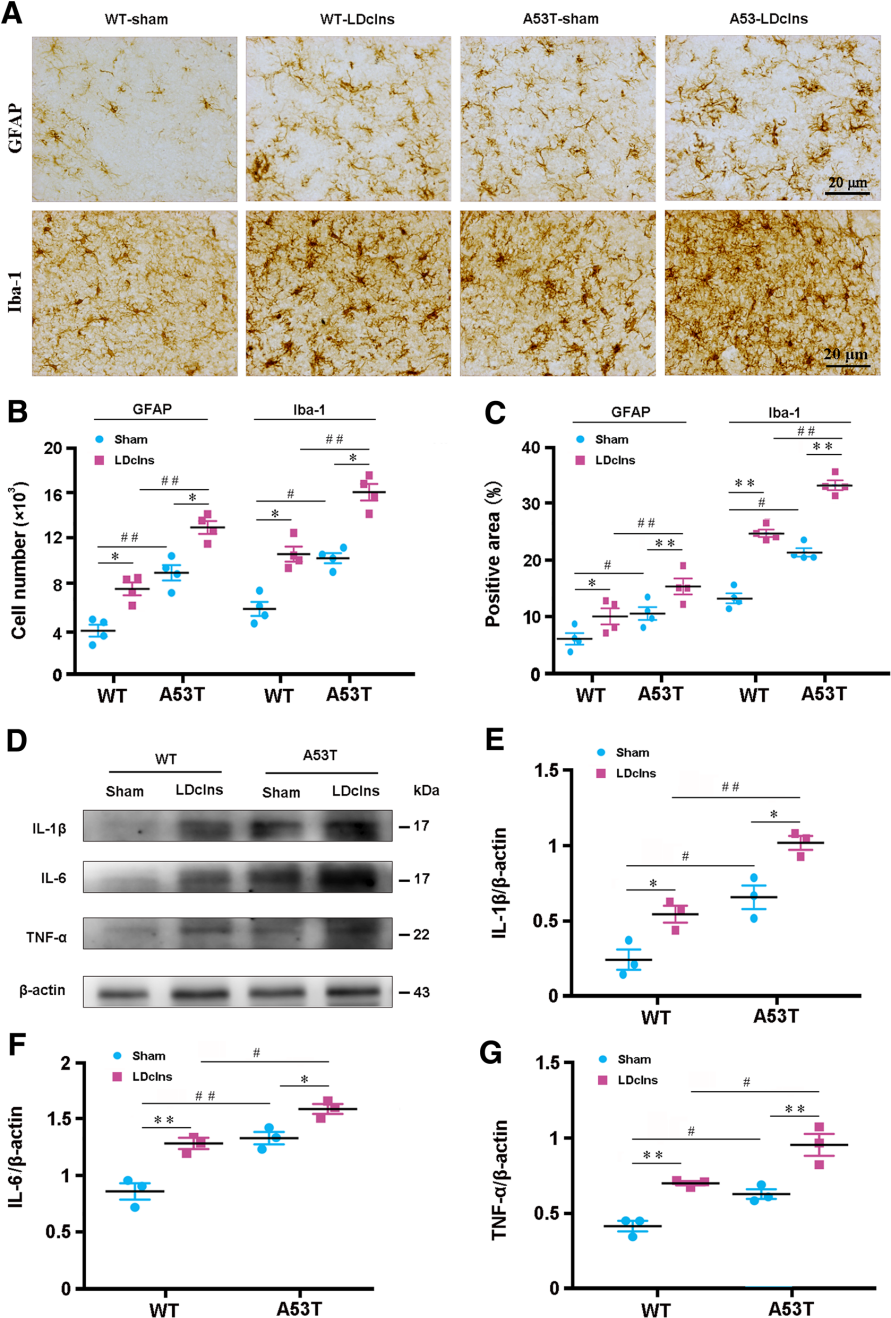
LDclns induced glial activation and inflammatory factor production in A53T mice and WT mice. **a** Representative micrographs showed activation of GFAP positive astrocytes and Iba-1 positive microglia in SN of A53T mice and WT mice that had Ldclns. **b** Stereological counts of GFAP positive cells (genotype, F_(1,12)_ = 44.98, *p* < 0.0001; ligament, F_(1,12)_ = 29.69, *p* = 0.0001; interaction, F_(1,12)_ = 0.0173, *p* = 0.8974) and Iba-1 positive cells (genotype, F_(1,12)_ = 26.97, *p* = 0.0002; ligament, F_(1,12)_ = 44.94, *p* < 0.0001; interaction, F_(1,12)_ = 0.5831, *p* = 0.4599) in SN of A53T-LDclns mice. **c** Percentage of positive area for GFAP (genotype, F_(1,12)_ = 18.54, *p* = 0.0010; ligament, F_(1,12)_ = 21.86, *p* = 0.0005; interaction, F_(1,12)_ = 0.0012, *p* = 0.9732) and Iba-1 (genotype, F_(1,12)_ = 36.02, *p* < 0.0001; ligament, F_(1,12)_ = 63.02, *p* < 0.0001; interaction, F_(1,12)_ = 0.5401, *p* = 0.4765) in SN. **d**-**g** Representative immunoblotting bands and densitometry analysis of IL-1β, IL-6 and TNF-α of ventral midbrain samples. Levels of IL-1β (genotype, F_(1,8)_ = 49.97, *p* = 0.0001; ligament, F_(1,8)_ = 27.86, *p* = 0.0007; interaction, F_(1,8)_ = 0.2273, *p* = 0.6463), IL-6 (genotype, F_(1,8)_ = 47.23, *p* = 0.0001; ligament, F_(1,8)_ = 36.64, *p* = 0.0003; interaction, F_(1,8)_ = 2.186, *p* = 0.1776) and TNF-α (genotype, F_(1,8)_ = 28.19, *p* = 0.0007; ligament, F_(1,8)_ = 48.21, *p* = 0.0001; interaction, F_(1,8)_ = 0.2262, *p* = 0.6471) were increased in both A53T mice and WT mice after LDclns. Data represent mean ± SEM from 3 to 4 mice per group; data in **e**-**g** are from two independent experiments

Previous studies showed that excessive aggregation of α-syn induces glial activation, subsequently causing release of inflammatory cytokines, and eventually intensifying degeneration of dopaminergic neurons [36, 37]. In the present study, activated GFAP-positive astrocytes and Iba-1 positive microglia were observed in WT-LDclns mice and A53T-sham mice, and more prominently in A53T-LDclns mice (Fig. 4a). Quantitative analysis revealed that both LDclns and transgenic α-syn increased number and area percentage of GFAP positive astrocytes as well as Iba-1 positive microglial cells (Fig. 4b, c). In line with reactive gliosis, fiercer neuroinflammatory response was present in SN of A53T-LDclns mice. IL-1β was increased by 187 and 154%, IL-6 was increased by 122 and 120%, and TNF-α was increased by 135 and 155% in A53T-LDclns mice, when compared with those in WT-LDclns mice and A53T-sham mice respectively (Fig. 4d-g).

### Ldclns aggravates impairment of AQP4 polarization in SN of A53T mice

**Fig. 5:**
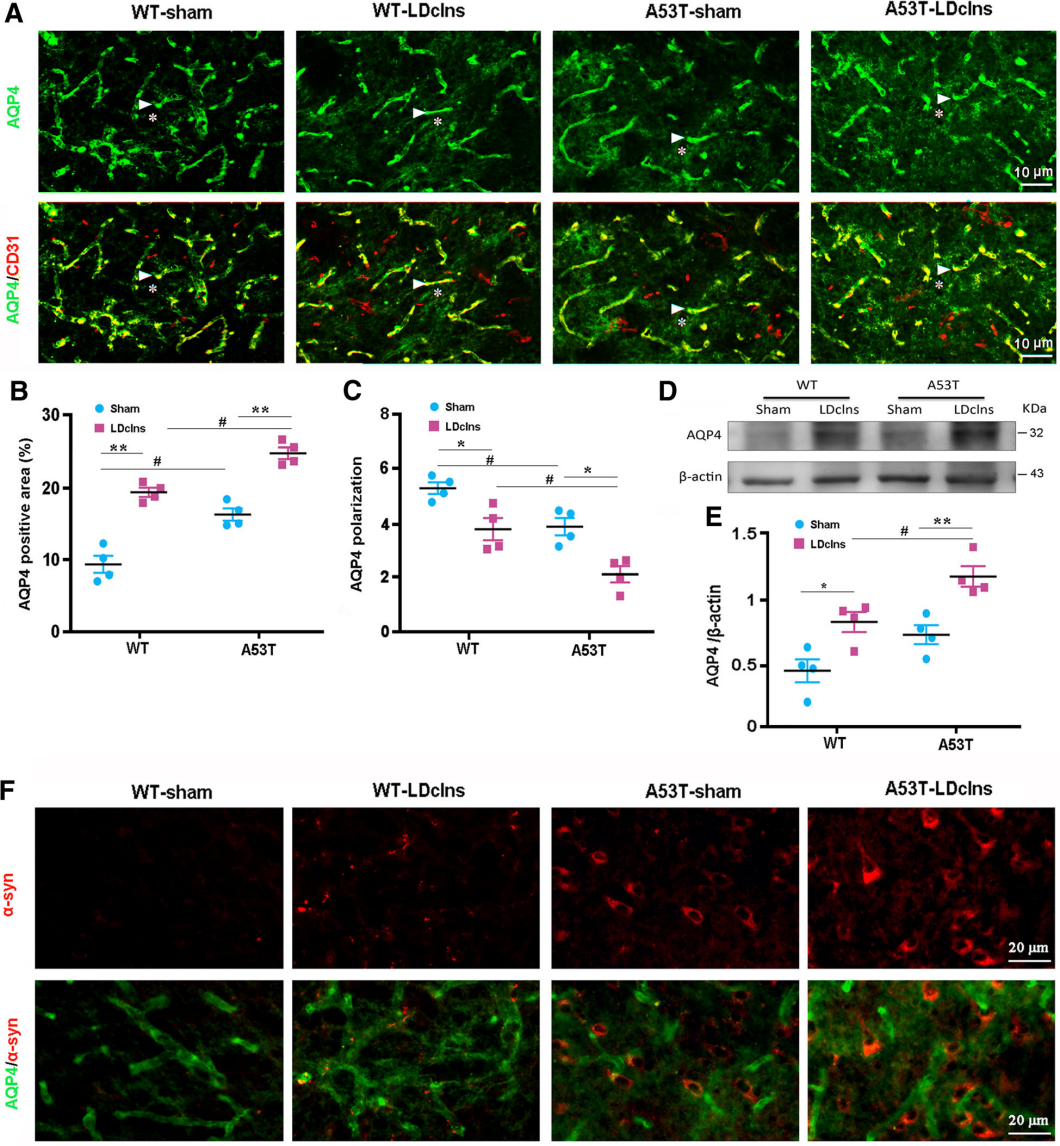
AQP4 depolarization was worsened in midbrain of A53T mice following blocking of meningeal lymphatics. **a** Immunofluorescence showed that AQP4 was restrictively expressed at astrocyte endfeet (arrowheads) surrounding CD31 positive microvessels in SN of WT-sham mice, but AQP4 immunoreactive signals were abnormally present at the neuropil domains (stars) of the other three groups, especially in A53T mice that had Ldclns. **b** Percentage of AQP4 positive area significantly increased in both WT-LDclns mice and A53T-LDclns mice (genotype, F_(1,12)_ = 32.82, *p* < 0.0001; ligament, F_(1,12)_ = 46.37, *p* < 0.0001; interaction, F_(1,12)_ = 0.1126, *p* = 0.7430). **c** AQP4 polarization, a ratio of perivascular AQP4 immunointensity to neutrophil AQP4 immunointensity, was lower in A53T-LDclns mice than WT-LDclns mice and A53T-sham controls respectively (genotype, F_(1,12)_ = 23.4, *p* = 0.0004; ligament, F_(1,12)_ = 26.22, *p* = 0.0003; interaction, F_(1,12)_ = 0.1357, *p* = 0.7191). **d**-**e** Representative immunoblotting bands and densitometry analysis of AQP4 expression from ventral midbrain samples. Levels of AQP4 were increased in both A53T mice and WT mice after LDclns (genotype, F_(1,12)_ = 15.69, *p* = 0.0019; ligament, F_(1,12)_ = 27.04, *p* = 0.0002; interaction, F_(1,12)_ = 0.2051, *p* = 0.6587). **f** Representative images showed the distribution pattern of AQP4 and α-syn. Notably, dense AQP4 immunoreactivity was closely surrounding α-syn positive cells. Data represent mean ± SEM from 4 mice per group; data in **e** are from two independent results

AQP4 is highly localized to perivascular endfeet, which is necessary for glymphatic solute transport in rodent brains [38–40]. Several studies from independent groups have confirmed that reactive astrogliosis causes loss of polarized AQP4 localization in astrocyte endfeet, subsequently impairing glymphatic clearance function [41–43]. We investigated whether reactive astrogliosis also affects AQP4 expression pattern in A53T mice with or without LDclns. Perivascular localization of AQP4 was present in SN of WT-sham mice. However, AQP4 was abnormally localized to the parenchymal regions of SN in the other three groups, particularly in A53T-LDclns mice, indicating impaired polarization of AQP4 (Fig. 5a). Quantitative data further confirmed changes of AQP4 expression in A53T-LDclns mice, with a marked increase in percentage of AQP4 positive area and decreases in AQP4 polarization, relative to WT-LDclns mice or A53T-sham mice (all *p* < 0.05; Fig. 5b, c). Furthermore, Western blotting showed that blocking meningeal lymphatics increased AQP4 expression in ventral midbrain of both mouse genotypes (*p* < 0.05, WT-sham vs WT-LDclns; *p* < 0.01, A53T-sham vs A53T-LDclns; Fig. 5d, e). Interestingly, α-syn positive neurons were closely surrounded by AQP4, indicating a correlation between depolarization of AQP4 and α-syn accumulation (Fig. 5f).

### AQP4 deletion impairs clearance of interstiatal α-syn from the parenchyma

**Fig. 6:**
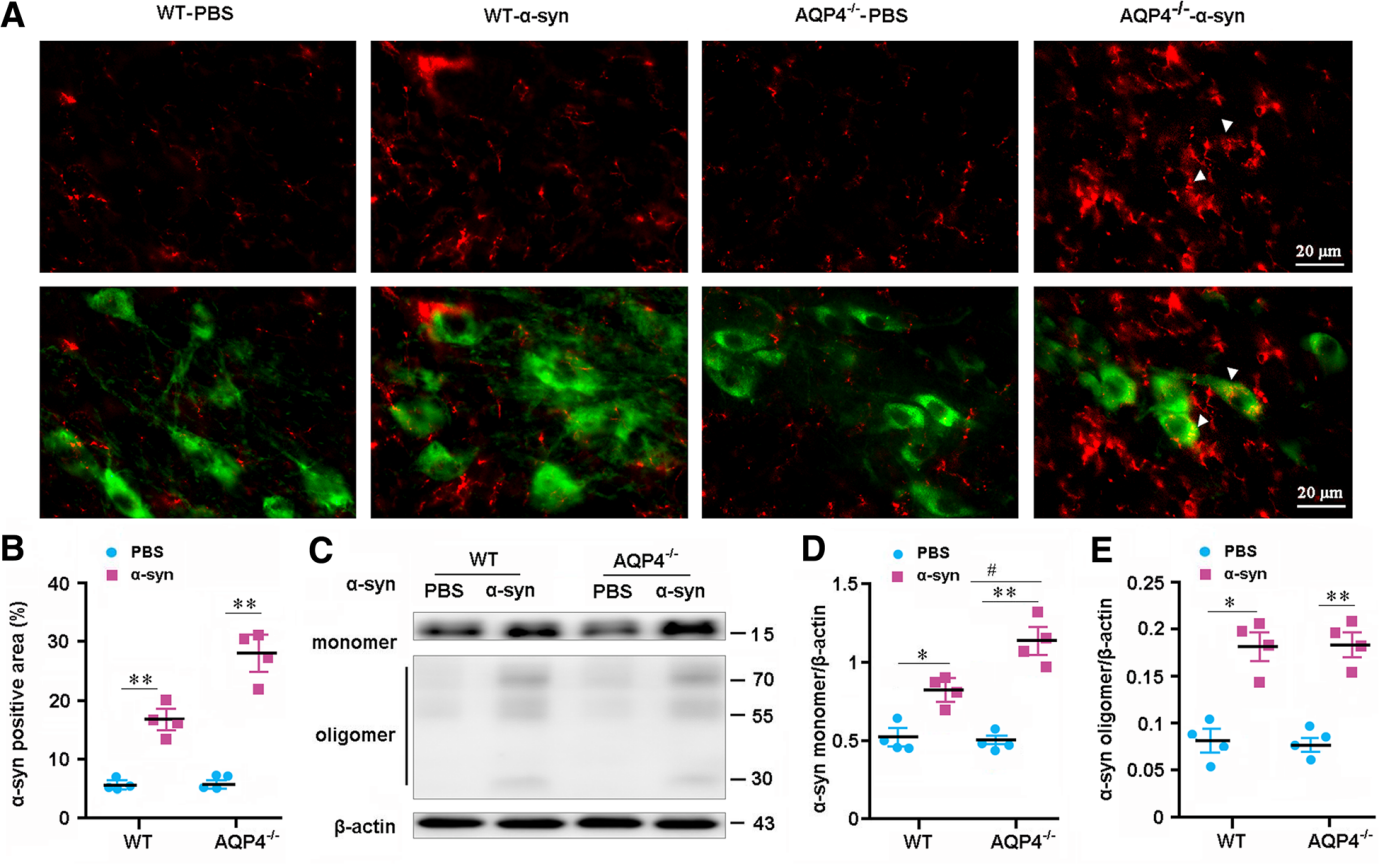
Deletion of AQP4 decreased clearance of soluble recombinant human α-syn A53T from the brain. **a** Representative micrographs showing α-syn immunoreactive products in SN of WT mice and AQP4^−/−^ mice at 2 h after injection of soluble human recombinant α-syn into SN. There was more obvious α-syn residue within SN of AQP4^−/−^ mice than WT mice. Importantly, in AQP4^−/−^ mice, α-syn immunoreactive signals were present within cytoplasm of TH-positive neurons (arrowhead), indicating existence of neuronal uptake of exogenous α-syn. **b** Percentage of α-syn positive area in SN was lower in WT mice than AQP4^−/−^ mice (genotype, F_(1,12)_ = 19.126, *p* = 0.001; ligament, F_(1,12)_ = 159.366, *p* < 0.0001; interaction, F_(1,12)_ = 18.037, *p* = 0.001). **c**-**e** Representative blotting bands and densitometry analysis of α-syn monomer and oligomers (monomer: genotype, F_(1,12)_ = 7.809, *p* = 0.016; injection, F_(1,12)_ = 80.394, *p* < 0.0001; interaction, F_(1,12)_ = 10.132, *p* = 0.008. oligomers: genotype, F_(1,12)_ = 0, *p* = 1; injection, F_(1,12)_ = 93.161, *p* < 0.0001; interaction, F_(1,12)_ = 0.065, *p* = 0.804) at 2 h after injection of α-syn. Data represent mean ± SEM from 4 mice per group. Data in **e** and **f** are from two independent experiments

To confirm AQP4 facilitating glymphatic clearance of α-syn, we investigated the effect of Aqp4 gene deletion on clearance of exogenously injected soluble recombinant human α-syn A53T and α-syn A30P from the brain parenchyma. Immunofluorescence showed that there was more α-syn residue in SN of AQP4^−/−^ mice at 2 h after intrastriatal injection of human α-syn (*p* < 0.05, versus WT; Fig. 6a, b; Additional file 5: Figure S5a-b). Western blot also revealed that AQP4^−/−^ brains contained a high residual level of soluble α-syn monomeric protein at 2 h after injection, relative to WT controls (*p* < 0.05; Fig. 6c, d). AQP4 deletion in mice did not increase aggregation of insoluble α-syn oligomeric protein following PBS or α-syn injection (Fig. 6c, e). This supports the view that AQP4 specifically mediates clearance of soluble macromolecules from the brain parenchyma [14, 15]. Consistently, ELISA results demonstrated that the rate of α-syn clearance was reduced in AQP4^−/−^ midbrain (both *p* < 0.05, versus WT control at 60 min and 120 min after injection; Additional file 5: Figure S5c). In addition, Western blotting showed that neither AQP4 deletion nor interstiatal injection of α-syn affected expression levels of autophagy markers LC3II/LC3I and p62 (Additional file 6: Figure S6a-c). Together, our data extends early results reporting that AQP4-dependent bulk flow facilitates clearance of ISF solutes, including Aβ and Tau from the brain [13].

### LDclns causes dopaminergic neuronal loss and motor impairments of A53T mice

**Fig. 7:**
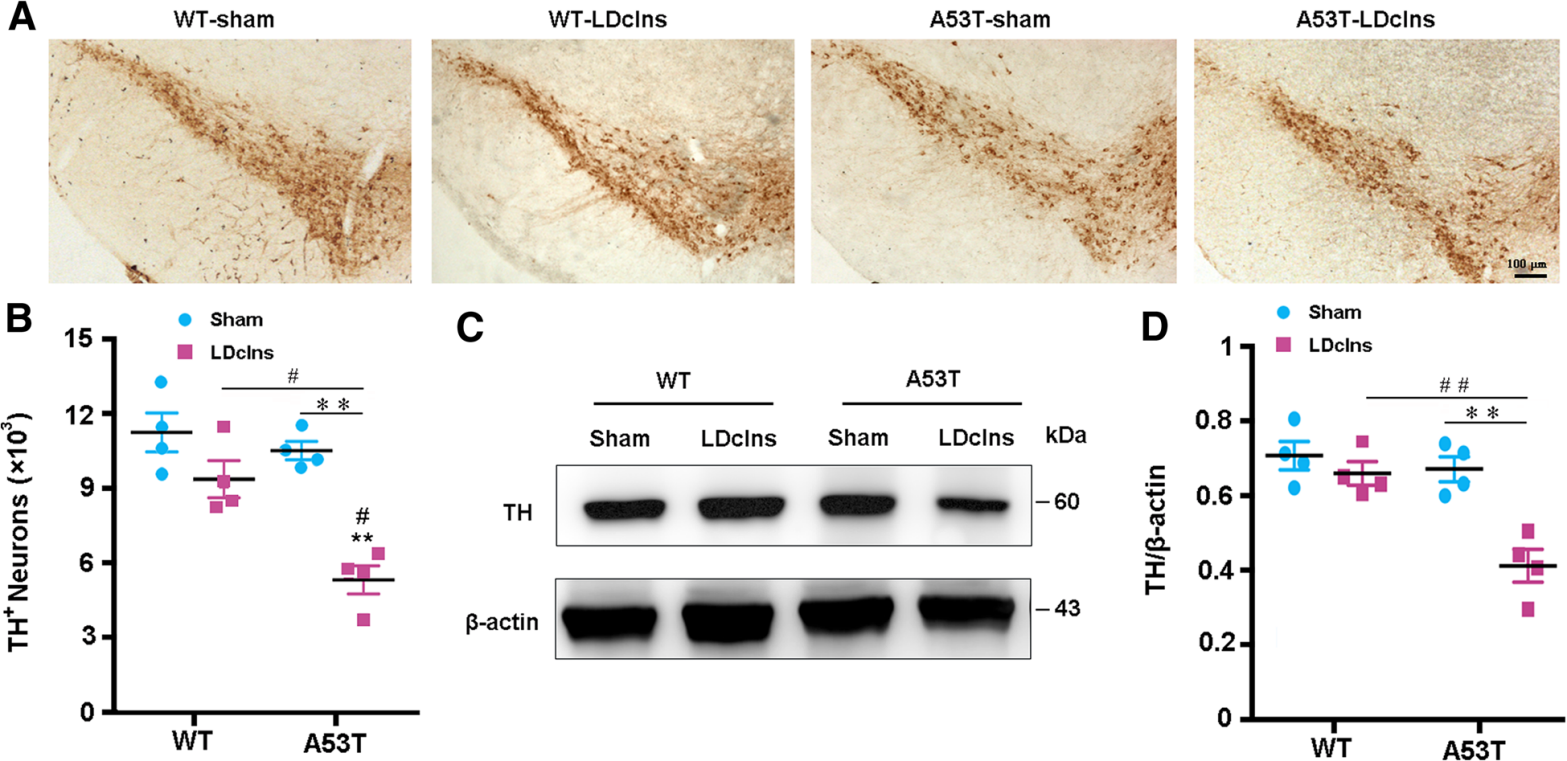
LDclns induced dopaminergic neuron loss in A53T mice. **a** Immunohistochemical staining of TH-positive neurons in SNc of mice. **b** Stereological counts revealed that numbers of TH-positive neurons significantly decreased in A53T-LDclns mice (genotype, F_(1,12)_ = 5.959, *p* = 0.0311; ligament, F_(1,12)_ = 13.35, *p* = 0.0033; interaction, F_(1,12)_ = 3.724, *p* = 0.0776). **c**-**d** Representative immunoblotting bands and densitometry analysis of TH expression that significantly decreased in A53T mice after LDclns (genotype, F_(1,12)_ = 14.70, *p* = 0.0024; ligament, F_(1,12)_ = 17.26, *p* = 0.0013; interaction, F_(1,12)_ = 8.222, *p* = 0.0142). Data represent mean ± SEM from 4 mice per group; data in **d** are from two independent experiments

We determined whether increased α-synuclein accumulation and secondary inflammatory response exacerbated the loss of dopaminergic neurons in A53T-LDclns mice by stereological counting of TH positive neurons throughout SN. There was no significant reduction of TH positive cells in A53T-sham mice compared with WT-sham mice (*p* > 0.05), but after LDclns, prominent loss of dopaminergic neurons occurred in A53T mice (*p* < 0.01; LDclns vs A53T-sham; Fig. 7a, b). Western blotting also confirmed downregulated expression of TH in A53T-LDclns mice (*p* < 0.01 vs A53T-sham; Fig. 7c, d).

**Fig. 8:**
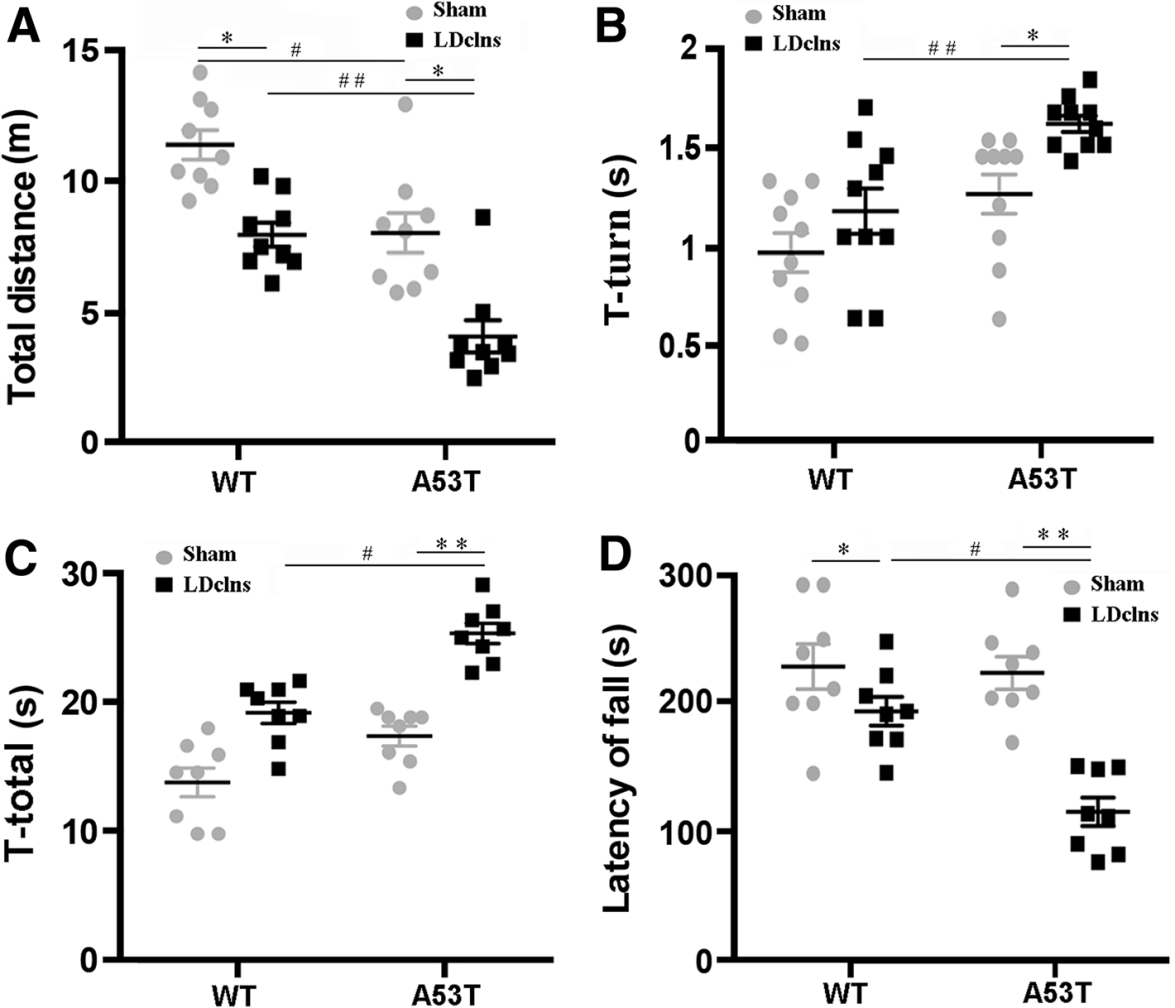
LDclns induced motor impairments in A53T mice. **a** Locomotor activity of mice was assessed with open-field test. The movement distance significantly decreased in LDclns-A53T mice (genotype, F_(1,32)_ = 17.87, *p* = 0.0002; ligament, F_(1,32)_ = 19.35, *p* = 0.0001; interaction, F_(1,32)_ = 0.1551, *p* = 0.6963). **b**-**c** Pole test was used to evaluate motor coordination of mice, showing that T-turn (genotype, F_(1,32)_ = 13.98, *p* = 0.0007; ligament, F_(1,32)_ = 11.01, *p* = 0.0023; interaction, F_(1,32)_ = 2.022, *p* = 0.1647) and T-total (genotype, F_(1,32)_ = 19.92, *p* < 0.0001; ligament, F_(1,32)_ = 24.10, *p* < 0.0001; interaction, F_(1,32)_ = 0.7967, *p* = 0.3788) significantly increased in A53T-LDclns mice. **d** Rotarod testing was also used to evaluate motor coordination of mice, the latency to fall off from the rod decreased significantly only in A53T-LDclns mice (genotype, F_(1,32)_ = 5.425, *p* = 0.0263; ligament, F_(1,32)_ = 17.38, *p* = 0.0002; interaction, F_(1,32)_ = 4.848, *p* = 0.035). Data represent mean ± SEM from 9 mice per group

We further investigated the consequences of LDclns on motor activity and coordination of A53T mice. At baseline, A53T mice showed normal motor coordination parameters including T-turn and T-total in the pole test and latency to fall in the rotoard (all *p* > 0.05; Fig. 8b-d), although total distance in the open-field test decreased, when compared with those in WT mice (*p* < 0.05; Fig. 8a). By contrast, total distance and latency decreased in A53T-LDclns mice, while T-turn and T-total increased when compared with A53T-sham mice (*p* < 0.01; *p* < 0.05; *p* < 0.01; *p* < 0.01, respectively) and WT-LDclns mice (*p* < 0.05; *p* < 0.01; *p* < 0.05; *p* < 0.05, respectively) (Fig. 8a-d).

## Discussion

A53T mice transgenically expressing mutant human α-syn can simulate the progressive pathological process of PD with appearance of motor impairments from 7 to 8 months old [43–45]. The present study found that 24-week old A53T mice do not show obvious abnormal behavioral performance in the pole test and rotoard test at baseline, despite a slight decline in total distance of movement during the open field testing when compared with WT mice. These A53T mice also do not show a significant loss of midbrain dopaminergic neurons. However, α-syn positive inclusion bodies are observed within neurons including TH-positive cells, suggesting that intracellular aggregation of α-syn is one of the early pathological events in this PD mouse model. Furthermore, the present results have revealed extracellular aggregation of α-syn especially in the perivascular space of SN in A53T mice. Impaired paravascular influx of CSF tracer further suggests that dysfunctions of the glymphatic clearance occur in the early stage of PD-like pathology. The decrease of the glymphatic clearance is mainly due to AQP4 mislocalization that caused by activated astrocytes, to be discussed later.

In addition, studies on human postmortem brains and cellular/animal models of PD have suggested a close interrelation between α-syn and autophagy [46–49]. Autophagy facilitates α-syn degradation and its impairment may favor α-syn inclusion formation [46]. On the other hand, accumulation of oligomeric α-syn is thought to be cytotoxic and damaging autophagy-lysosomal function subsequently, resulting in a pathological cycle leading to neuronal death [49]. The present results show that blocking meningeal lymphatic drainage alters expression levels of autophagy marker LC3II/LC3I in A53T mice but not in WT mice. Likewise, the level of insoluble α-syn only increases in A53T-ligated mice. These data suggest that intercellular autophagy degradation may play a compensatory role in clearance of α-syn in the healthy brain when CNS lymphatic drainage is blocked. However, this compensatory mechanism is insufficient due to a long-term over-production of α-syn combined with meningeal lymphatic dysfunction. Taken together, the present results suggest that decline of glymphatic clearance is involved in the early pathological process of A53T transgenic PD mouse model. This finding supports the view that the glymphatic system has an essential role in maintaining metabolism homeostasis of macromolecules in the brain and its alteration is involved in the pathogenesis of macromolecule aggregation related neurodegeneration [16, 50].

Recent studies have identified meningeal lymphatic vessels in human and rodent brains [51, 52]. They align with dural blood vessels and exit the cranium via the foramina together with venous sinuses, arteries and cranial nerves [53, 54]. Functional studies have revealed that meningeal lymphatic vessels mainly contribute to the delivery of CSF macromolecules within the subarachnoid space into the Dclns [11, 16, 17, 55, 56]. For example, Da Mesquita and colleagues (2018) have demonstrated that disruption of meningeal lymphatic vessels decreases paravascular influx of macromolecules into the brain and efflux of macromolecules from the ISF in young-adult mice [16]. Our results show that intracisternal fluorescent tracer drainage into the Dclns is almost completely abrogated after blocking their afferent lymphatic vessels. Our results also demonstrate that astrocytes with damaged AQP4 polarity are activated and the levels of α-syn and tau proteins are increased in the midbrain of WT-LDclns mice. Furthermore, blocking meningeal lymphatic drainage in WT mice leads to an increase in inflammatory cytokines accompanied with mild dysfunctions of motor and coordination. Similarly, early studies reported that ablation of Dclns results in cognitive impairment, which is associated with disruption of the normal flow of meningeal T-cells into the Dclns [57]. We have demonstrated that numbers of CD3^+^, CD4^+^ and CD8^+^ T cells are significantly increased in the meninges, but decreased in the Dclns after ligation of their afferent lymphatic vessels. These findings together with previous studies highlight the functional communications between the glymphatic system and extracranial lymphatic drainage pathway [15–17, 56, 58].

LDclns significantly aggravates α-syn pathology in A53T mice. As mentioned above, there is no obvious motor abnormality in 24-week old A53T mice compared to WT controls. However, A53T mice that received LDclns show significant dysfunctions of coordination and balance that evidenced by decreases in the total distance of spontaneous movement in the open field and the time of staying on the rotarod, and increases in T-turn and T-total in the pole test. Consistent with behavioral alterations, blocking meningeal drainage aggravates glymphatic clearance dysfunction causing worse deposition of α-syn, glial activation, inflammation, and dopaminergic neuronal loss. Our results strongly suggest that overexpression of α-syn leads to reactive astrogliosis, which impairs AQP4 polarity and in turn hampers glymphatic clearance function. However, a considerable proportion of excessive α-syn can still be cleared from the brain parenchyma due to functional preservation of extracranial lymphatic drainage thereby alleviating the PD-like pathological process in A53T mice.

**Fig. 9:**
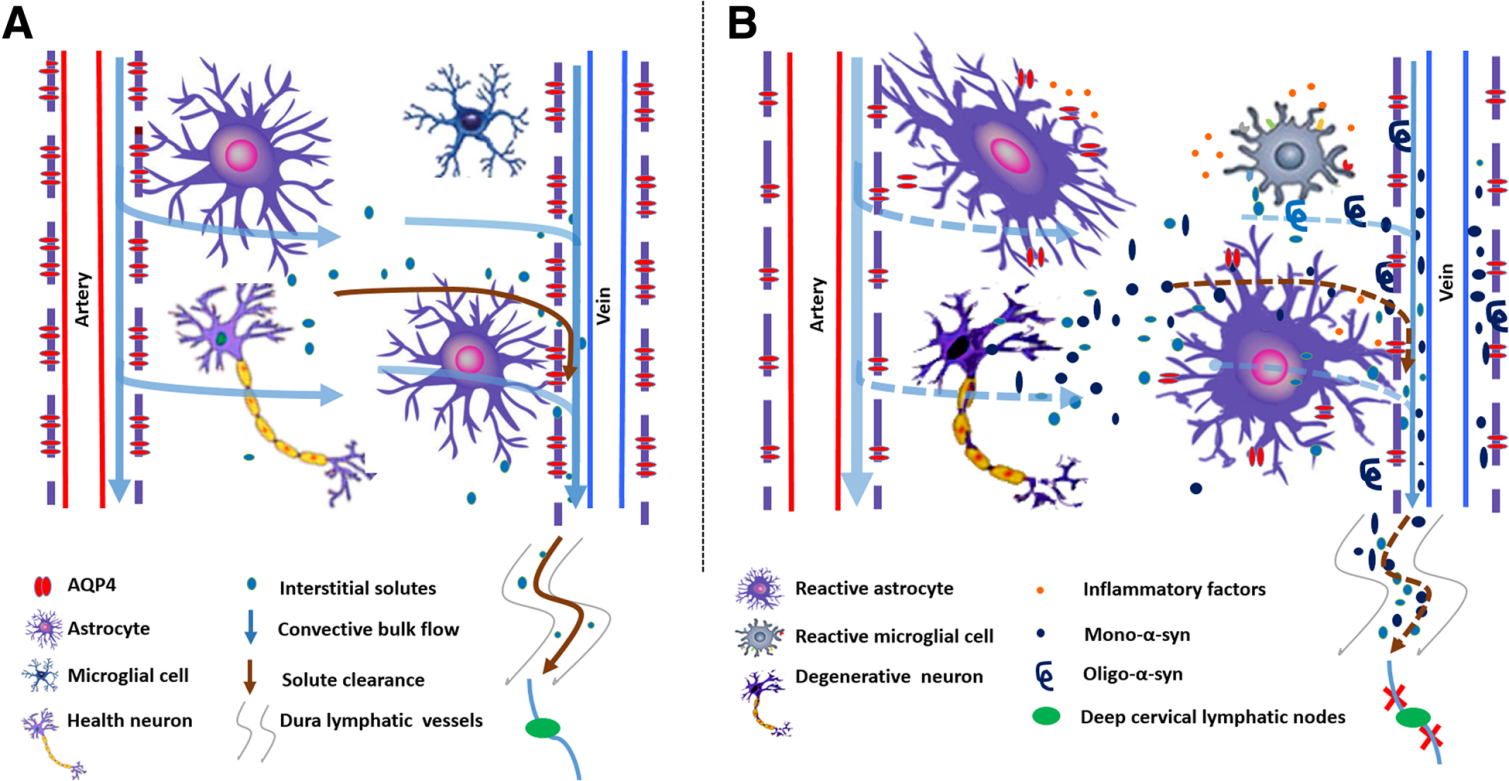
Schematic diagram of aggravated PD-like pathological progression in A53T mice following blocking brain lymphatic drainage. **a** In intact condition, AQP4-dependent astroglial water transport facilitates convective bulk flow along para-arterial space-interstitial space-par-venous space routes, which drives clearance of interstitial solutes from the brain parenchyma to enter the subarachnoid CSF, and subsequently transport into meningeal lymphatic vessel-cervical lymph node pathway. **b** Astrocyte activation caused by excessive production of α-syn impairs AQP4 polarity surrounding microvessels, which reduces glymphatic clearance and subsequently leads to gradual aggregation of α-syn with age in A53T mice. LDclns disrupts meningeal lymphatic clearance of α-syn, further aggravating the above vicious circle and causing dopaminergic neuronal degeneration and motor malfunction

Under intact conditions, high expression of AQP4 at the perivascular endfeet of astrocytes mediates rapid CSF-ISF movement from the peri-arterial space into the interstitium and subsequently into the perivenous space of deep draining veins, facilitating clearance of brain metabolites and toxic solutes (Fig. 9a) [56]. Several groups independently reported that astrocyte AQP4 is essential for fast glymphatic transport [13, 59–63], although there is literature not supporting this view [64]. In response to a variety of damage stimuli, astrocytes are activated with mislocalization of AQP4 thereby impairing the normal flow direction of CSF-ISF and corresponding solute clearance [28]. Indeed, loss of perivascular AQP4 polarization has been reported in various animal models including aging [28, 62], AD [65], traumatic brain injury [66] and subarachnoid hemorrhage [67]. In addition, the efflux of radiolabeled mannitol and Aβ in brain were reduced in AQP4^−/−^ mice with slowed glymphatic flow [13]. Consistently, the present results show that AQP4 deficiency decreases interstiatal α-syn clearance from the brain parenchyma. Our previous study also showed that knocking out the Aqp4 gene exacerbates brain Aβ deposition and cerebral amyloid angiopathy in APP/PS1 mice [27]. Studies in human specimens also report that Aβ deposition is associated with aberrant AQP4 expression in aging brains [68]. This suggests that AQP4 polarized localization at the endfeet of astrocytes is necessary for glymphatic clearance. Furthermore, Hoshi and colleagues reported numerous AQP4-positive astrocytes in the neocortex of patients with PD and a significant negative correlation between the levels of AQP4 and a-syn in layers V–VI of neocortex [69]. Targeting AQP4 is expected to be new strategies for the treatment of neurodegenerative diseases associated with aggregation of toxic metabolites.

In summary, we have showed that blocking extracranial lymphatic drainage markedly exacerbates accumulation of α-syn in the brain parenchyma of A53T mice and subsequently leads to extensive reactive astrogliosis and AQP4 polarity destruction, thus exacerbating glymphatic clearance deficits and promoting α-syn-related pathology (Fig. 9b). However, it should be noted that the lymphatic system is rich in collateral circulation, likely after LDclns, compensatory effects of other extracranial lymphatic systems including the cribriform plate route need to be analyzed. The results suggest that both intracellular degradation and extracellular clearance of α-syn are impaired in A53T mice, but which one is an initial event needs to be further explored. In addition, pathogenic point mutations in the α-syn gene, such as A53T and A30P missense mutations, are linked to familial PD. However, most neurodegenerative disorders involving Lewy bodies are associated with abnormal accumulation of wild-type α-syn [3]. Neuronal over-expression of wild-type human α-syn in mice resulted in progressive accumulation of α-syn in neurons, associated with loss of dopaminergic terminals in the basal ganglia and with motor impairment [70]. Whether brain lymphatic clearance dysfunction is also involved in pathology of this PD model and patients with AD warrants further investigation.

## Conclusions

Our results demonstrate an impairment of glymphatic clearance of α-syn in the brain of A53T mice, and disruption of meningeal lymphatic drainage further exacerbates α-syn-related pathology. Findings suggest that lymphatic clearance dysfunction is crucial in the onset and development of PD, and might be therapeutically targeted to alleviate age-associated neurodegenerative disorders.

## Additional files

Additional file 1: **Figure S1.** The long-term consequence of LDclns on histological profile of Dclns. HE staining demonstrated that the majority of cells in the ligated lymph nodes were not lymphocytes but fibroblasts instead. (TIF 2052 kb)

Additional file 2: **Figure S2.** Analysis of T cells distribution in the meninges and dcLNs after blocking meningeal lymphatic drainage. a-b Gating strategy and representative dot plots for CD3^+^ T cells (a), CD4^+^ T cells and CD8^+^ cells (b) in the Dclns and meninges of A53T mice. c-d Percentage of CD3^+^ T cells, CD4^+^ T cells and CD8^+^ cells in the meninges (c) and Dclns respectively (d). All data represent mean ± SEM from 4 mice per group from two independent experiments. Student-t test, *p < 0.05, **p < 0.01, ***p < 0.001, sham vs LDclns. (TIF 4540 kb)

Additional file 3: **Figure S3.** LDclns exacerbated the accumulation of tau and PHF-1 in A53T mice. a Representative bands of Western blotting of Tau and PHF-1. b-c Densitometry analysis showed that expression levels of Tau were significantly increased in A53T-LDclns (genotype, F_(1,8)_ = 28.54, *p* = 0.0007; ligament, F_(1,8)_ = 29.36, *p* = 0.0006; interaction, F_(1,8)_ = 0.04947, *p* = 0.8296) and expression levels of PHF-1 were significantly increased in A53T-LDclns (genotype, F_(1,8)_ = 43.72, *p* = 0.0002; ligament, F_(1,8)_ = 28.88, *p* = 0.0007; interaction, F_(1,8)_ = 0.05677, *p* = 0.8177). Data represent mean ± SEM from 3 mice per group from two independent experiments. The statistical analysis was performed by two-way ANOVA, followed by the Tukey’s post hoc test. *p < 0.05, **p < 0.01, DClns vs Sham; # *p* < 0.05, ## *p* < 0.01 A53T vs WT. (TIF 1243 kb)

Additional file 4: **Figure S4.** Analyses of brain water content after LdcLNs. Brain water content was not affected by genotype (F_(1,12)_ = 2.540, *p* = 0.1370), ligament (F_(1,12)_ = 1.589, *p* = 0.2315) nor their interaction (F_(1,12)_ = 0.08899, *p* = 0.7706). Data represent mean ± SEM from 4 mice per group. (TIF 936 kb)

Additional file 5: **Figure S5.** Deletion of AQP4 decreased clearance of interstitial soluble human recombinant α-syn A30P from the brain. **a** Representative micrographs showing α-syn immunoreactive products in SN of WT mice and AQP4^−/−^ mice at 2 h after injection of α-syn A30P into SN. A considerable proportion of α-syn immunoreactive signals was observed within cytoplasm of TH-positive neurons of AQP4^−/−^ mice (arrowhead). b Percentage of α-syn positive area in SN was lower in WT mice than AQP4^−/−^ mice (genotype, F_(1,12)_ = 58.91, *p* < 0.0001; injection, F_(1,12)_ = 14.85, *p* = 0.0023; interaction, F_(1,12)_ = 2.018, *p* = 0.1809). c ELISA analysis of α-syn in ventral midbrain samples. Over the first 2 h after injection, clearance of α-syn from AQP4^−/−^ mouse brains was significantly reduced, compared to WT controls. Data represent mean ± SEM from 4 mice per group (b) and per time point (c). Data in c are from two independent experiments. Statistical analysis was performed by two-way ANOVA, followed by the Tukey’s post hoc test (b) or Student-t test (c). *p < 0.05, **p < 0.01, α-syn vs PBS; # *p* < 0.05, ## *p* < 0.01, AQP4^−/−^ vs WT. (TIF 7402 kb)

Additional file 6: **Figure S6.** Analysis autophagy in ventral midbrain of WT and AQP4 mice following injection of PBS or soluble recombinant human α-syn into SN. a-c Representative immunoblotting bands and densitometry analysis of LC3 and p62. The ratio of LC3II/LC3I and levels of p62 were not affected by genotype (F_(1,12)_ = 0.065, *p* = 0.803; F_(1,12)_ = 0.045, *p* = 0.836, respectively), injection (F_(1,12)_ = 0.007, *p* = 0.934; F_(1,12)_ = 0.301, *p* = 0.593, respectively) or their interaction (F_(1,12)_ = 0.261, *p* = 0.619; F_(1,12)_ = 0, *p* = 1, respectively). Data represent mean ± SEM from 4 mice per group from two independent experiments. (TIF 3161 kb)
