# Supplementary figures and images for: Blocking meningeal lymphatic drainage aggravates Parkinson’s disease-like pathology in mice overexpressing mutated α-synuclein
Source: Transl Neurodegener. 2019 Mar 1;8:7. doi: 10.1186/s40035-019-0147-y (PMC6396507; doi:10.1186/s40035-019-0147-y)

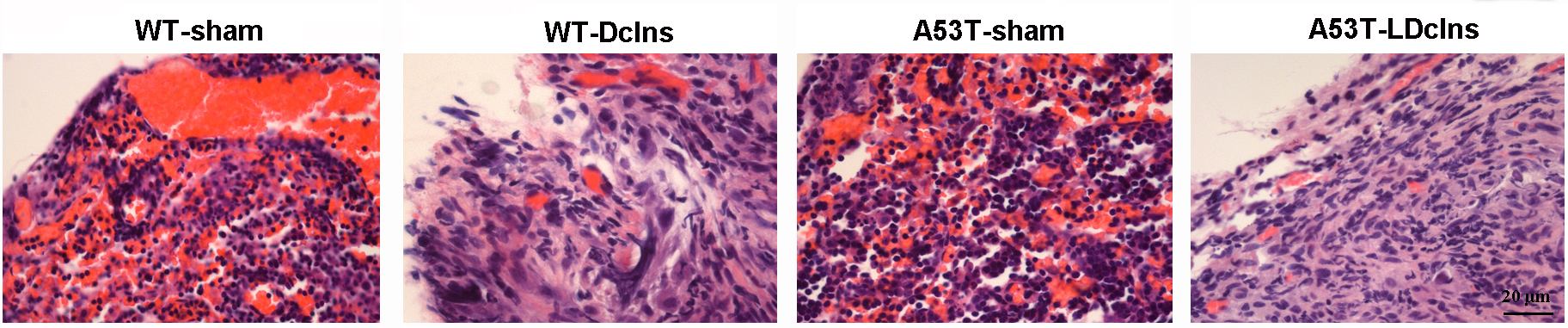

Supplement: Supplementary file 1 — Figure S1. The long-term consequence of LDclns on histological profile of Dclns. HE staining demonstrated that the majority of cells in the ligated lymph nodes were not lymphocytes but fibroblasts instead. (TIF 2052 kb) [file 40035_2019_147_MOESM1_ESM.tif]

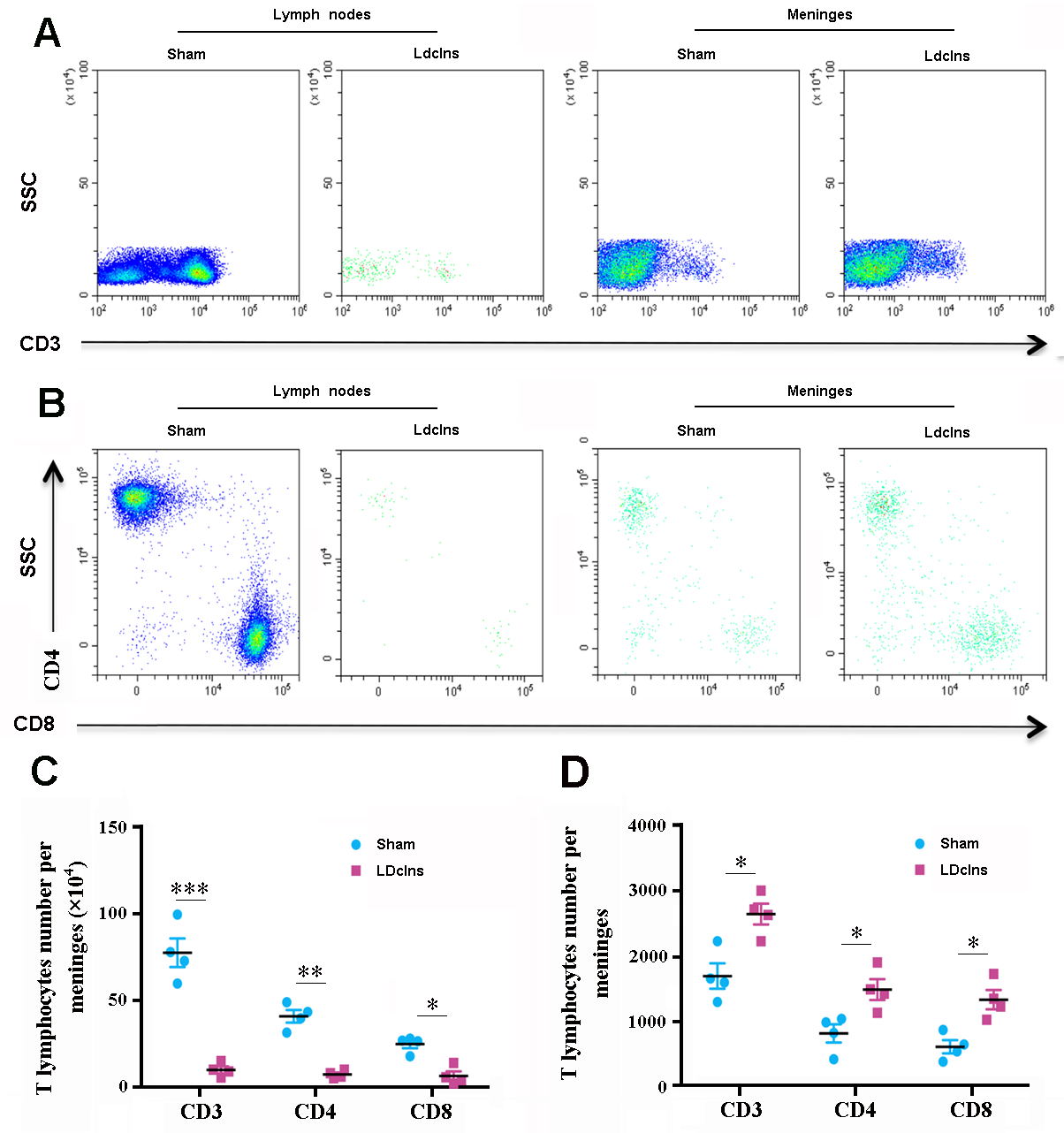

Supplement: Supplementary file 2 — Figure S2. Analysis of T cells distribution in the meninges and dcLNs after blocking meningeal lymphatic drainage. a-b Gating strategy and representative dot plots for CD3+ T cells (a), CD4+ T cells and CD8+ cells (b) in the Dclns and meninges of A53T mice. c-d Percentage of CD3+ T cells, CD4+ T cells and CD8+ cells in the meninges (c) and Dclns respectively (d). All data represent mean ± SEM from 4 mice per group from two independent experiments. Student-t test, *p < 0.05, **p < 0.01, ***p < 0.001, sham vs LDclns. (TIF 4540 kb) [file 40035_2019_147_MOESM2_ESM.tif]

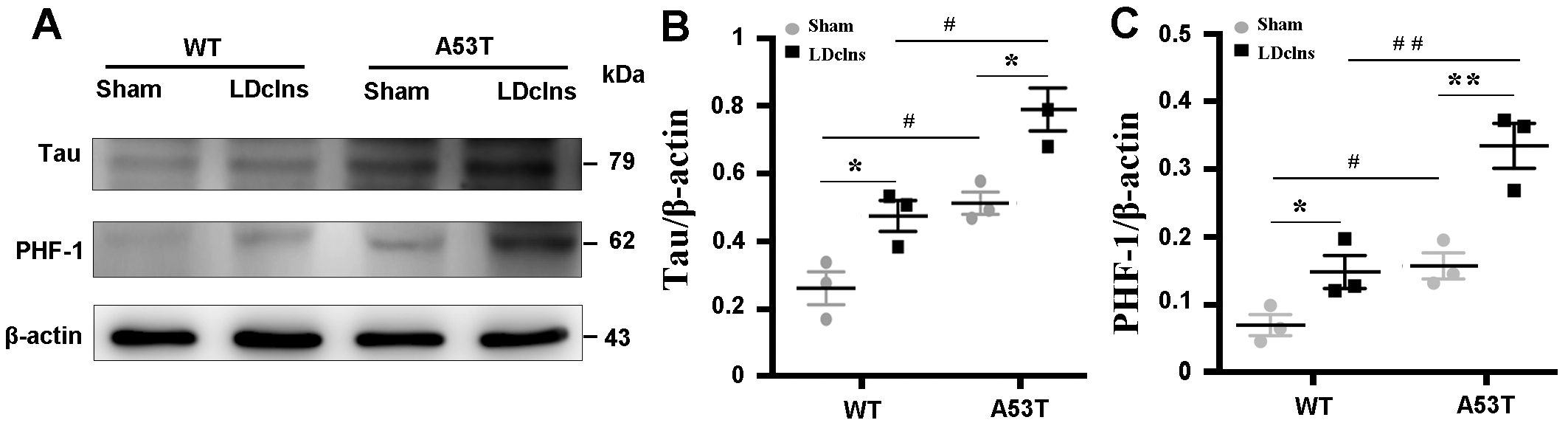

Supplement: Supplementary file 3 — Figure S3. LDclns exacerbated the accumulation of tau and PHF-1 in A53T mice. a Representative bands of Western blotting of Tau and PHF-1. b-c Densitometry analysis showed that expression levels of Tau were significantly increased in A53T-LDclns (genotype, F(1,8) = 28.54, p = 0.0007; ligament, F(1,8) = 29.36, p = 0.0006; interaction, F(1,8) = 0.04947, p = 0.8296) and expression levels of PHF-1 were significantly increased in A53T-LDclns (genotype, F(1,8) = 43.72, p = 0.0002; ligament, F(1,8) = 28.88, p = 0.0007; interaction, F(1,8) = 0.05677, p = 0.8177). Data represent mean ± SEM from 3 mice per group from two independent experiments. The statistical analysis was performed by two-way ANOVA, followed by the Tukey’s post hoc test. *p < 0.05, **p < 0.01, DClns vs Sham; # p < 0.05, ## p < 0.01 A53T vs WT. (TIF 1243 kb) [file 40035_2019_147_MOESM3_ESM.tif]

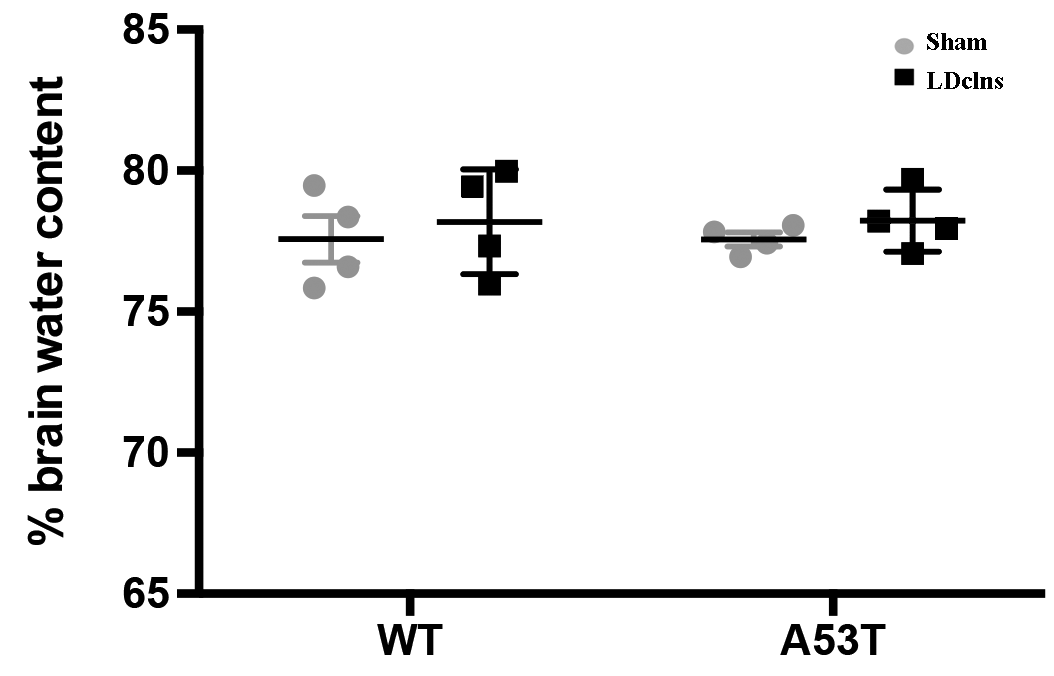

Supplement: Supplementary file 4 — Figure S4. Analyses of brain water content after LdcLNs. Brain water content was not affected by genotype (F(1,12) = 2.540, p = 0.1370), ligament (F(1,12) = 1.589, p = 0.2315) nor their interaction (F(1,12) = 0.08899, p = 0.7706). Data represent mean ± SEM from 4 mice per group. (TIF 936 kb) [file 40035_2019_147_MOESM4_ESM.tif]

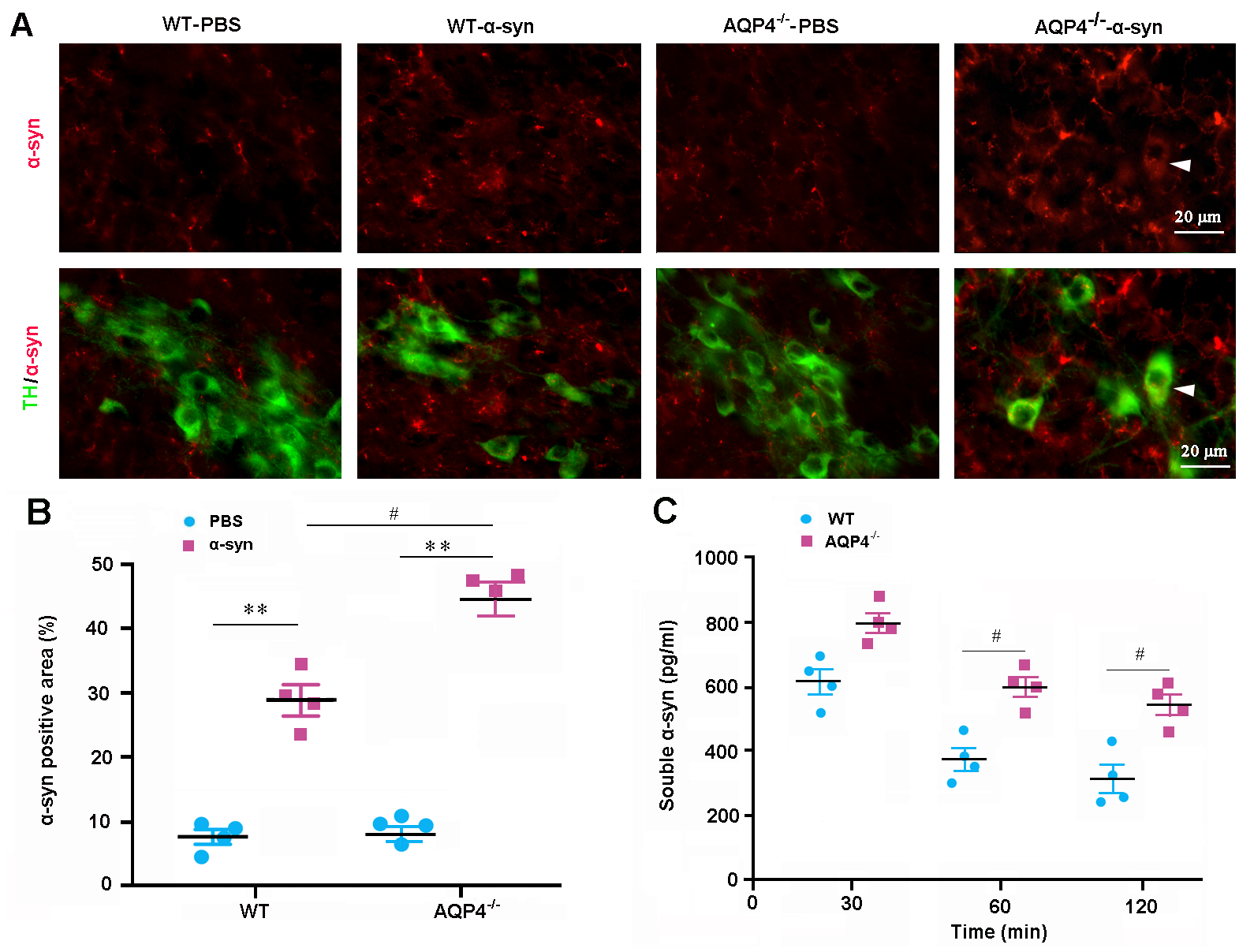

Supplement: Supplementary file 5 — Figure S5. Deletion of AQP4 decreased clearance of interstitial soluble human recombinant α-syn A30P from the brain. a Representative micrographs showing α-syn immunoreactive products in SN of WT mice and AQP4−/− mice at 2 h after injection of α-syn A30P into SN. A considerable proportion of α-syn immunoreactive signals was observed within cytoplasm of TH-positive neurons of AQP4−/− mice (arrowhead). b Percentage of α-syn positive area in SN was lower in WT mice than AQP4−/− mice (genotype, F(1,12) = 58.91, p < 0.0001; injection, F(1,12) = 14.85, p = 0.0023; interaction, F(1,12) = 2.018, p = 0.1809). c ELISA analysis of α-syn in ventral midbrain samples. Over the first 2 h after injection, clearance of α-syn from AQP4−/− mouse brains was significantly reduced, compared to WT controls. Data represent mean ± SEM from 4 mice per group (b) and per time point (c). Data in c are from two independent experiments. Statistical analysis was performed by two-way ANOVA, followed by the Tukey’s post hoc test (b) or Student-t test (c). *p < 0.05, **p < 0.01, α-syn vs PBS; # p < 0.05, ## p < 0.01, AQP4−/− vs WT. (TIF 7402 kb) [file 40035_2019_147_MOESM5_ESM.tif]

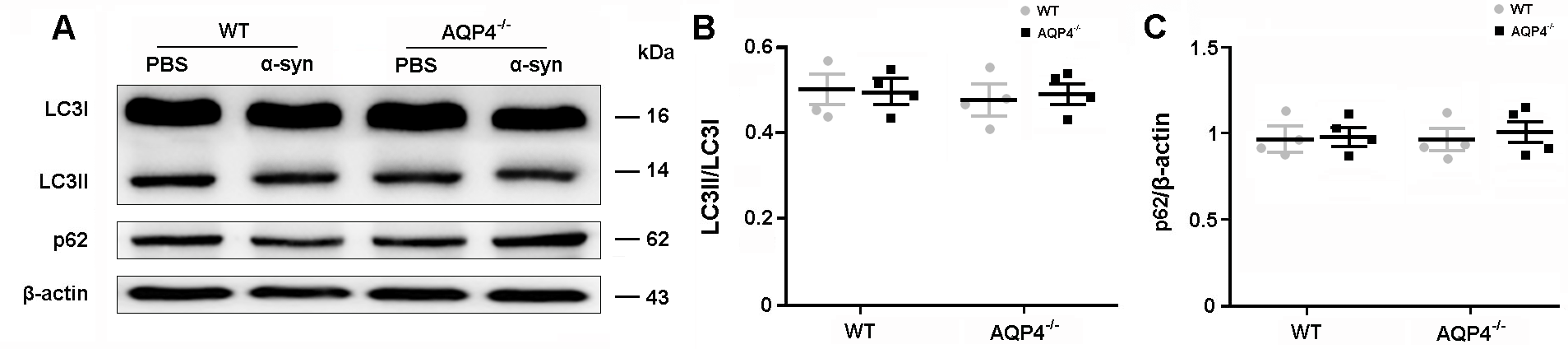

Supplement: Supplementary file 6 — Figure S6. Analysis autophagy in ventral midbrain of WT and AQP4 mice following injection of PBS or soluble recombinant human α-syn into SN. a-c Representative immunoblotting bands and densitometry analysis of LC3 and p62. The ratio of LC3II/LC3I and levels of p62 were not affected by genotype (F(1,12) = 0.065, p = 0.803; F(1,12) = 0.045, p = 0.836, respectively), injection (F(1,12) = 0.007, p = 0.934; F(1,12) = 0.301, p = 0.593, respectively) or their interaction (F(1,12) = 0.261, p = 0.619; F(1,12) = 0, p = 1, respectively). Data represent mean ± SEM from 4 mice per group from two independent experiments. (TIF 3161 kb) [file 40035_2019_147_MOESM6_ESM.tif]
